# Antimicrobial resistance at the human–animal interface in the Pastoralist Communities of Kasese District, South Western Uganda

**DOI:** 10.1038/s41598-020-70517-w

**Published:** 2020-09-07

**Authors:** Jacob Stanley Iramiot, Henry Kajumbula, Joel Bazira, Catherine Kansiime, Benon B. Asiimwe

**Affiliations:** 1 Department of Medical Microbiology, College of Health Sciences, Makerere University School of Biomedical Sciences, P.O Box 7072, Kampala, Uganda; 2 Department of Microbiology and Immunology, Faculty of Health Sciences, Busitema University, Mbale, Uganda; 3 Department of Microbiology, Faculty of Medicine, Mbarara University of Science and Technology, Mbarara, Uganda

**Keywords:** Antimicrobials, Bacteria, Bacteriology, Microbiology, Medical research

## Abstract

Intensive usage of antimicrobials in the management of animal diseases leads to selection for resistance among microorganisms. This study aimed to assess antimicrobial use and to describe factors associated with the transmission of antimicrobial resistance between humans and animals in pastoralist communities of Kasese district. A mixed-methods approach was employed in this study. Rectal swabs were collected from the participants and cattle and transported in Carry–Blaire transport medium to the laboratory within 24 h of collection for culture and sensitivity to confirm carriage of multi-drug resistant bacteria. In-depth interviews were conducted among veterinary officers, veterinary drug vendors, human health facility in-charges in both public and private health facilities, and operators of human pharmacies and drug shops. Carriage of multi-drug resistant bacteria among humans was 88 (93%) and 76(80%) among cattle. Consumption of lakeshore water and carriage of multi-drug resistant bacteria in cattle were associated with carriage of multi-drug resistant bacteria in the human population. The prevalence of multi-drug resistance among organisms Isolated from both humans and animals was high. There is a high likelihood of transmission of multi-drug resistance between humans and animals.

## Introduction

Intensive usage of antimicrobials in the management of animal diseases may cause selection for resistance among microorganisms^1^. Transmission of resistant bacteria from the wild and domestic food animals to humans may occur via the food chain, environment, or direct interaction with animals and this may lead to the emergence of infections that are challenging to manage^2^. It is now estimated that antimicrobial resistance (AMR) among bacteria, viruses, or causes 700,000 deaths annually^3^ hence posing a significant public health challenge across the world. The resistant clones of bacteria have gained a global distribution with over 90% being resistant to commonly used antibiotics such as co-trimoxazole, penicillin, ampicillin, and gentamicin among others^4^. Most human diseases originate from animals with 61% being zoonotic^5^. Bacteria from animals are among the predominant causes of diseases in the food chain^2^. Even with the recognition of these facts, zoonotic infections frequently remain undiagnosed in humans and usually mistaken for other febrile diseases such as malaria among others^6,7^. The present success of animal husbandry has been linked to the use of antibiotics as growth promoters and in the prevention of infections but this may present a huge cost for the future. Humans have never been healthier, wealthier, or more numerous but this may not be the same in the future as the microbial world presents the important challenge of antimicrobial resistance and a reversal to the pre-antibiotic era^8^. Whereas in theory, zoonotic infections are best controlled in the animal host, in practice, pastoralist communities are often left to manage disease themselves using inherent knowledge with the focus on treatment rather than prevention^8^. While the One-health concept aims to attain optimal health for humans, animals, and the environment, this approach is largely still theoretical in developing countries such as Uganda. In Queen Elizabeth National Park, many vectors feed on both the wild and domestic animals (cattle). The wild animals are seldom treated, if at all; the cattle on the other hand are all the time treated with antibiotics by the untrained pastoralists. There are few veterinary officers in this area of the country and the cost for their services usually inhibits the pastoralist communities from seeking their help and advice, they. The pastoralist communities have therefore taken the driving seat of antimicrobial resistance through indiscriminate administration of antimicrobial agents often in sub-optimal doses. This study aimed to assess antimicrobial use and to describe factors associated with transmission of antimicrobial resistance between humans and animals in pastoralist communities of Kasese district.

## Methods

### Study area and setting

The study was conducted in and around Queen Elizabeth National Park (QENP), Western Uganda. The Kasese side of the National Park has two pastoralist communities in Nyakatonzi and Hima sub-countries. The QENP lies astride the equator along the latitudes of 0 Ê 39′ 36″ North, 30 Ê 16′ 30″ East. QENP is located in the western part of Uganda on the floor of the western arm of the East African Rift Valley. QENP forms part of an extensive trans-boundary ecosystem that includes Kibale National Park to the northeast, Rwenzori Mountains National Park to the northwest, and is also contiguous with Virunga National Park in the Democratic Republic of Congo^9,10^.

### Study population and sampling

**Figure 1:**
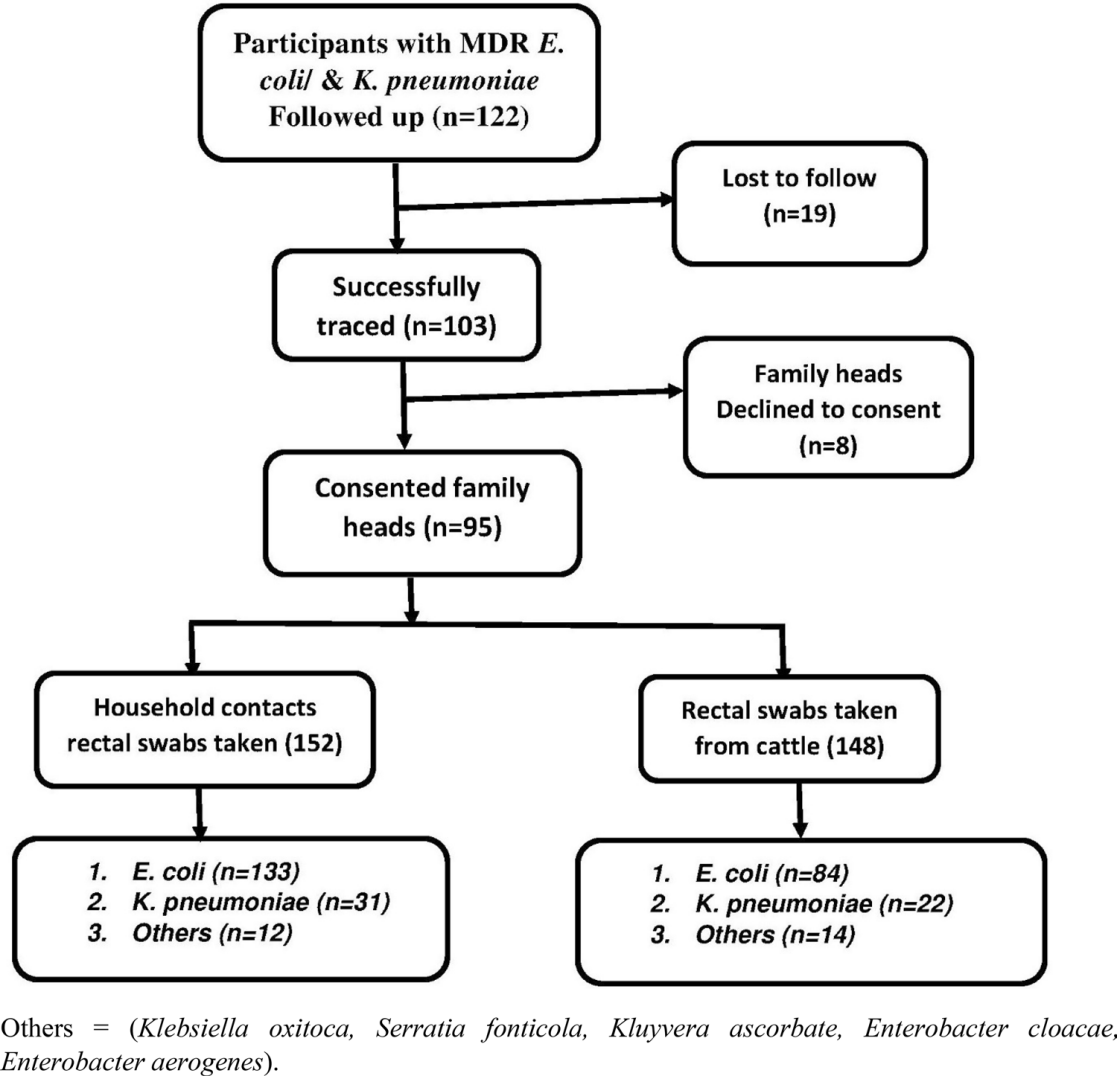
Study profile. Others = (Klebsiella oxitoca, Serratia fonticola, Kluyvera ascorbate, Enterobacter cloacae, Enterobacter aerogenes).

The study population comprised of pastoralist communities of Kasese district and their cattle. Rectal swabs were collected from 152 humans and 148 cattle (Fig. 1) and transported in the Carry–Blaire transport medium to the laboratory within 24 h of collection. Sample processing, culture, and subsequent tests were performed at the Clinical Microbiology Laboratory, Department of Medical Microbiology, Makerere University College of Health Sciences. A questionnaire was then administered to the respondents who were above 18 years to assess factors associated with carriage of multidrug resistance. The key informants were selected based on a range of characteristics: their role in the health facility/pharmacy/drug shop and administrative positions occupied that directly affect the use of antimicrobials. The investigator contacted all the key informant interviewees on the phone or in-person to schedule appointments for the interviews.

### The study design

A mixed-methods approach was employed in this study between January 2018 and March 2019. A total of 122 participants who previously tested positive for multi-drug resistant *E.* *coli* were followed up to the community (Fig. 1). In the community, questionnaires were administered to the traced patients/parents/guardians who consented to take part in the study to assess antibiotic consumption both in animals and humans, self-medication, and other variables as per the conceptual framework. Presence or absence of multidrug-resistant isolates were our outcome variables while the predictor variables were cattle keeping, handling of cattle and their products, consumption of animal products, consumption of antimicrobial agents by both animals and humans, sanitation and social status, history of hospitalization in the household, education level, occupation, location of the household from the clinic and socio-demographic characteristics such as age and sex. Rectal swabs were then collected from two randomly selected members of the household, and two randomly selected animals (cattle) from each homestead using sterile cotton swabs for culture and sensitivity. The collected specimens were transported in the Carry-Blair transport medium to the Microbiology laboratory of Makerere University School of Biomedical Sciences for analysis. Speciation and antibiotic susceptibility of the isolates was done using the Phoenix automated microbiology system (Phoenix 100 ID/DST system) from Becton and Dickson (Franklin Lakes, NJ, USA) and the results interpreted using the CLSI guidelines.

In-depth interviews were conducted among the veterinary medicine practitioners including; Veterinary Officers and veterinary drug vendors, human health facility in-charges in both public and private health facilities and operators of human pharmacies and drug shops who were purposively selected due to their role in the health facility/pharmacy/drug shop and administrative positions occupied that directly affect the use of antimicrobials. The research assistants were trained to collect quality information at its depth. For convenience, appointments were scheduled with the prospective interviewees who met our selection criteria at the time which was most convenient to them and each interview lasted between 1 and 2 h. All the respondents were interviewed at their places of work to minimize the disruption of their normal routines. Before the commencement of the interview, the interviewer explained the aims and scope of the study and obtained written informed consent from every participant who accepted to take part in the study. Face-to-face interviews were conducted with the aid of an interview guide that was prepared by the investigator, reviewed by co-investigators, and refined as the final tool for data collection. All the 12 interviews were audio-recorded and later transcribed into manuscripts by the field research assistants for analysis.

### Data management and analysis

Data were coded and entered into Epi-Data version 3.1 software and later exported to STATA V14 for analysis. Categorical data were presented as proportions and their associations determined by the Chi-square test. Bivariate analysis was performed to explore associations between the isolation of multi-drug resistant bacteria and associated factors thereof. Variables were included in the multivariate analysis basing on factors in the bivariate analyses that had either *p* ≤ 0.2 and/or other variables such as the history of chronic illness known from the literature to be associated with the risk of acquiring multidrug resistance. In all cases, *P* ≤ 0.05 was considered as evidence of significant statistical association.

Data from the in-depth interviews were transcribed verbatim in the English language by the trained research assistants and thematic analysis as described before^11^ was used to inform data analysis. For quality control, transcription was done by the first research assistant and the second research assistant listened to the audio recording while reading the transcripts to recheck on the quality of the transcripts. Data analysis was done using the NVivo V12 qualitative data analysis software (QSR International (Americas) Inc). Emergent themes were identified, coded, and organized into concepts that were later developed into explanations. Data analysis involved a process of familiarization with the data by listening to the audio recordings and reading the transcript several times while noting ideas. The principle investigator performed the thematic content analysis and the co-investigators verified the themes and contents. Significant statements relating to antimicrobial resistance were identified and coded. The codes were clustered and themes were generated. Emergent themes generated were then discussed with co-investigators. The process of theme generation was reviewed and refined by going back and forth between the themes and codes, as well as between the themes and transcripts until the final themes were identified, defined and named. The final themes were discussed with some of the respondents in the process of member checking. All methods were performed according to the relevant guidelines and regulations.

### Ethical considerations

**Figure 2:**
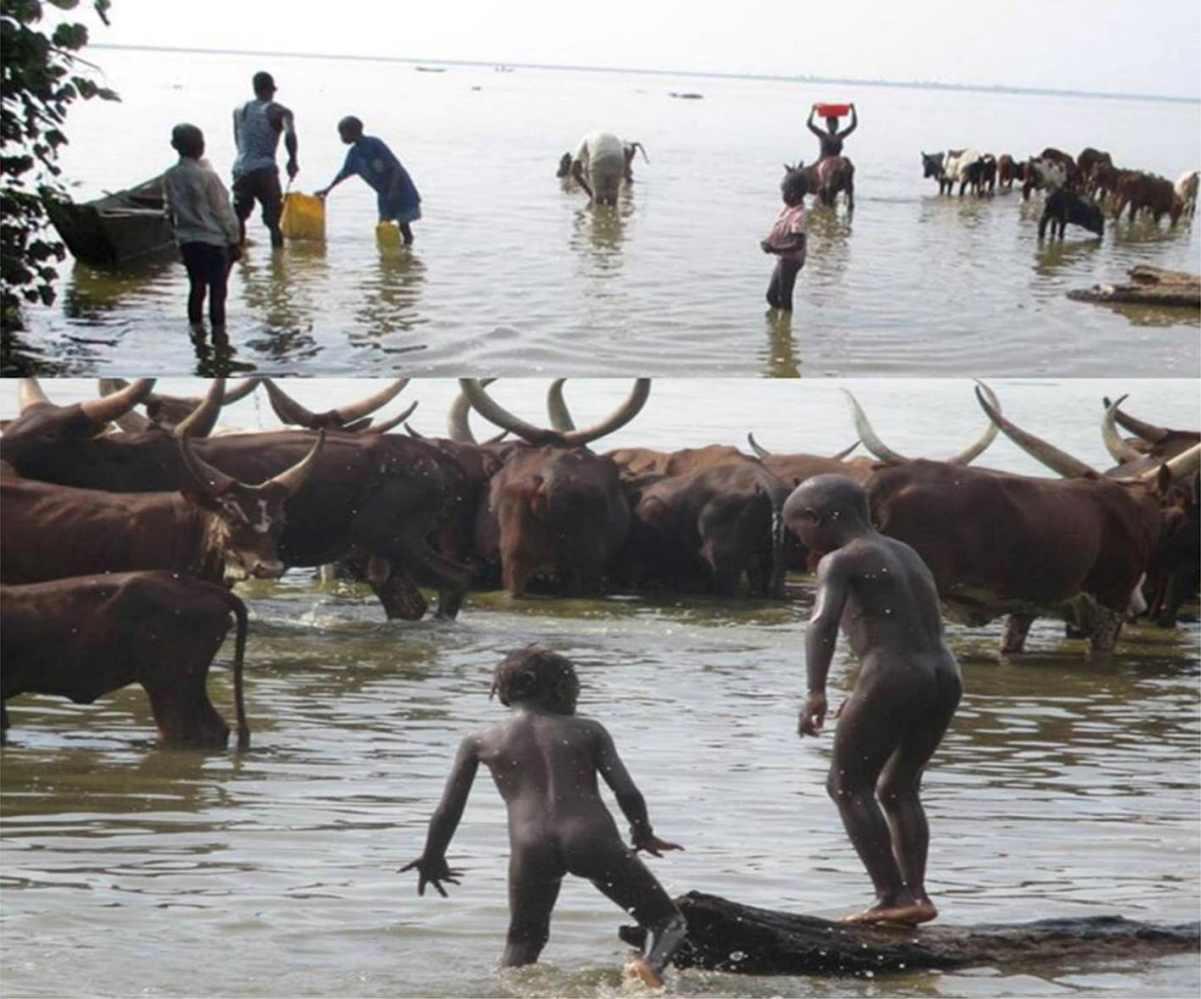
Routine activities in pastoralist communities. Children swimming in a landing site while cattle drink water and women fetch water for domestic chores.

The study was approved by the Makerere University School of Biomedical Sciences Higher Degrees and Ethics Committee (SBS-HDREC) and The Uganda National Council of Science and Technology (UNCST). Written informed consent was obtained from all respondents. The picture in Fig. 2 was taken from the backside after obtaining consent to ensure confidentiality.

 Approval to use animals in this study was obtained from the Uganda Wildlife Authority (UWA) and written informed consent to collect, store and use samples from animals/cattle for research was obtained from the owners of the animals. All methods used in this study were following the relevant standard guidelines and regulations.

## Results

Out of the 122 respondents who fulfilled our selection criteria for follow up to the community, 103 respondents were successfully traced, 19 were lost to follow, 95 consented to participate in the study, and 8 declined to participate with no specific reasons. The probable reason for a high number of the lost to follow was that at the time of follow up coincided with the cultivation season and many people had moved to the low lands to cultivate.

The mean age of the respondents was 35 years, the youngest participant was 18 years whereas the oldest was 66 years. Females constituted slightly over half, 55 (58%) of the total number of respondents.

The common livestock kept were cattle, goats, sheep, and pigs. The local breeds of livestock were generally preferred over the crossbreeds because they are resistant to diseases and can survive well under the local weather conditions. Crossbreeds are gaining acceptance among some pastoralists because they are high performers in terms of production. Cross-breeds grow very fast and they can be marketed before encountering a lot of expense of feeding because they are fast growers and the farmer can get quick returns. Pastoralists reported that that cross-breeds need a high level of management in terms of disease control yet drugs are very expensive.

### Knowledge, opinions, and perceptions on antimicrobial resistance

Respondents were requested to respond with ‘True’, ‘False’, or ‘I don’t know’ to a couple of questions to assess their knowledge, opinions, and perceptions on antimicrobial resistance. The majority of the respondents in this study 74 (78%) could not correctly define antimicrobial resistance. A high proportion of the respondents, 74 (78%) agreed with the statement that “antimicrobial resistance occurs when your body becomes resistant to antimicrobials and they no longer work well”. Many respondents also agreed that “antimicrobial resistance is an issue that could affect them or their families” 68 (72%), “can be transmitted from person to person, and very challenging to treat” 75 (79%) (Table 1).

**Table 1:** Knowledge, opinions, and perceptions of antimicrobial resistance.

Respondents’ responses on the understanding of the issue of antimicrobial resistance	True; No (%)	False; No (%)	I don’t Know; No (%)
(1) Antimicrobial resistance occurs when your body becomes resistant to antimicrobials and they no longer work as well	74 (78)	8 (8)	13 (14)
(2) Many infections are becoming increasingly resistant to treatment by antimicrobials	56 (59)	22 (23)	17 (18)
(3) If bacteria are resistant to antimicrobials, it can be very difficult or impossible to treat the infections they cause	75 (79)	7 (7)	13 (14)
(4) Antimicrobial resistance is an issue that could affect me or my family	68 (72)	14 (15)	13 (14)
(5) Antimicrobial resistance is an issue in other countries but not here	7 (7)	74 (78)	14 (15)
(6)Antimicrobial resistance is only a problem for people who take antimicrobials regularly	41 (43)	41 (43)	13 (14)
(7) Bacteria which are resistant to antimicrobials can be spread from person to person	75 (79)	8 (8)	12 (13)
(8) Antimicrobial-resistant infections could make medical procedures like surgery, organ transplants and cancer treatment much more dangerous	49 (52)	31 (33)	15 (16)

Opinion	Right; No (%)	Wrong; No (%)	No opinion; No (%)
**Do** **you** **feel** **the** **following** **actions** **would** **help** **address** **the** **problem** **of** **antimicrobial** **resistance?**			
(1) People should use antimicrobials only when they are prescribed by a doctor or nurse	90 (95)	4 (4)	1 (1)
(2) Pastoralists should give fewer antimicrobials to food-producing animals	84 (88)	10 (11)	1 (1)
(3) People should not keep antimicrobials and use them later for other illnesses	58 (61)	37 (39)	0 (0)
(4) Parents should make sure all of their children’s vaccinations are up-to-date	87 (93)	7 (7)	0 (0)
(5) People should wash their hands regularly	95 (100)	0 (0)	0 (0)
(6)Doctors should only prescribe antimicrobials when they are needed	92 (97)	2 (2)	1 (1)
(7)Governments should reward the development of new antimicrobials	62 (65)	32 (34)	1 (1)
(8)Pharmaceutical companies should develop new antimicrobials	63 (67)	31 (33)	0 (0)
(9)“It’s okay to use antimicrobials that were given to a friend or family member, as long as they were used to treat the same illness”	63 (66)	31 (33)	1 (1)

Which of the following conditions should antibiotics be used to treat	Yes	No	
(a) Malaria	55 (58)	40 (42)	
(b) Urinary tract infections	50 (53)	45 (47)	
(c) Respiratory tract infections	76 (80)	19 (20)	
(d) Fungal infections of the skin	23 (24)	72 (76)	
(e) Wound infections	47 (49)	48 (51)	
(f) Gonorrhea	63 (66)	32 (34)	
(g) Sore throats	79 (83)	16 (17)	
(h) Cold and flu	87 (92)	8 (8)	

The respondents agreed with several actions that would address the problem of antimicrobial resistance. Most of the respondents agreed that people should use antimicrobials only when they are prescribed by a doctor or nurse, 90(95%), parents should ensure all of their children’s vaccinations are up-to-date, 87 (93%) and doctors should only prescribe antimicrobials when they are needed 92 (97%). All respondents agreed that regular hand washing was key in minimizing the problem of antimicrobial resistance.

There were also several misconceptions among the respondents about the use of antibiotics. Over 50% reported that antibiotics can be used to treat malaria, respiratory tract infections 76 (80%) sore throat 79 (83%), and common cold 87 (92%).

Different socio-demographic factors such as education level, having heard about antimicrobial resistance, sources of drinking water, a recent visit to a health facility, level of health care facility visited, History of medical procedure, chronic conditions, over the counter access to antimicrobials, having had a procedure involving the use of Internal devices, frequency of antimicrobial use, multi-drug resistance in bacteria isolated from Cattle were analyzed using bivariate and multivariate analysis to determine their association with multi-drug resistance carriage among the isolated bacteria from humans (Table 2). Drinking lakeshore water and carriage of MDR bacteria in cattle were significantly associated with carriage of multi-drug resistant bacteria in humans (*P* < 0.05). None of the other socio-demographic characteristics was significantly associated with carriage of multi-drug resistant bacteria in humans.

**Table 2:** Factors Associated with MDR in bacteria Isolated in the pastoralists community of Kasese district.

Factors	No (%)	Presence of MDR No (%)	Absence of MDR No (%)	COR	AOR	95% CI	*P* *value*
**Gender**							
Male	40 (42)	39 (97)	1 (3)	4.7	7.6	0.7862–74.2152	0.08
Female	55 (58)	49 (89)	6 (11)				
**Education level **							
No formal education	19 (20)	18 (95)	1 (5)	0.75		0.3038–1.8751	0.54
Primary	40 (42)	36 (90)	4 (10)				
Secondary	30 (32)	30 (100)	0 (0)				
Tertiary	6 (6)	4 (67)	2 (33)				
**Heard of AMR**							
Yes	23 (24)	19 (83)	4 (17)	4.8	5.1	0.9390–28.6662	0.06
No	72 (76)	69 (96)	3 (4.2)				
**Source of water **							
Borehole	29 (31)	25 (86)	4 (14)	1.6		1.0150–2.5062	0.04
Protected spring	3 (3)	26 (67)	1 (33)				
Open well	1 (1)	1 (10)	0 (0)				
Lake shore	62 (65)	60 (97)	2 (3.2)				
**A recent visit to a hospital**							
2 weeks ago	11 (12)	11 (100)	0 (0)	0.6		0.3491–1.36566	0.29
1 month ago	11 (12)	11 (100)	0 (0)				
> 3 months ago	28 (30)	24 (86)	4 (14)				
> 6 months ago	30 (32)	29 (97)	1 (3)				
> 1 year ago	14 (15)	12 (86)	2 (14)				
**Health care level visited**							
HCII	33 (35)	33 (100)	0 (0)	0.8		0.5148–1.3943	0.51
HCIII	16 (17)	13 (81)	3 (19)				
HCIV	1 (1)	1 (100)	0 (0)				
Hospital	30 (32)	26 (87)	4 (13)				
Private clinic	15 (16)	15 (100)	0 (0)				
**History of medical procedure**							
Yes	12 (13)	10 (83)	2 (17)	0.3	0.5	0.0073–29.7543	0.7
No	83 (87)	78 (94)	5 (6)				
**Chronic conditions**							
Yes	34 (36)	32 (94)	2 (6)	1.4		0.2619–7.7916	0.68
No	61 (64)	56 (92)	5 (8)				
**Over the counter access to antimicrobials **							
Yes	41 (43)	37 (90)	4 (10)	0.5		0.1148–2.5781	0.44
No	54 (57)	51 (94)	3 (0.1)				
**Internal devices **							
Yes	9 (9)	7 (78)	2 (22.2)	0.2	0.1	0.0020–7.3649	0.3
No	86 (91)	81 (94)	5 (5.8)				
**Frequency of antimicrobial use**							
Rarely	26 (27)	26 (100)	0 (0)	0.6	0.8	0.2985–2.2464	0.7
Frequently	60 (63)	56 (93)	4 (6.7)				
Don’t know	9 (10)	6 (67)	3 (33.3)				
**MDR in cattle**							
Yes	76 (89)	74 (970	2 (2.6)	13		2.3276–75.0209	0.004
No	19 (11)	14 (74)	5 (26.3)				

Two hundred and ninety-six (296) bacteria isolated from both humans and animals were tested for resistance to a panel of 15 antibiotics belonging to different classes. The bacteria isolated included; *Escherichia* *coli,* *Klebsiella* *pneumoniae,* *Klebsiella* *oxitoca,* *Serratia* *fonticola,* *Kluyvera* *ascorbate,* *Enterobacter* *cloacae,* *and* *Enterobacter* *aerogenes.* The antibiotics used were selected from the penicillins, β-lactamase inhibitors, 1st, 2nd, 3rd, and 4th generation cephalosporins, quinolones/fluoroquinolones, aminoglycosides, tetracyclines, nitrofurantoins, carbapenems, and folate pathway inhibitors. Generally, the prevalence of antibiotic resistance among the isolated organisms was high, with a number showing multi-drug resistance.

Resistance against cefazolin, ampicillin, cotrimoxazole, and amoxicillin-clavulanic acid was high among the *E.* *coli* isolates from humans (98, 85, 72, 60) % respectively (Table 3). However, gentamicin, ciprofloxacin, levofloxacin, and imipenem showed low resistance patterns (2, 5, 5, 5) %, respectively. Among Klebsiella isolates, resistance was high against cefazolin, amoxicillin-clavulanic acid, cefuroxime, and cefepime (94, 87, 74, 61) %, respectively whereas low resistance patterns against ciprofloxacin, levofloxacin, imipenem, and ertapenem (1, 1, 2, 4) % respectively were observed. None of the Klebsiella isolated from humans was resistant to gentamicin. Other isolates (Table 3) were resistant to ampicillin, cefazolin, and cefuroxime and also exhibited high resistance patterns to amoxicillin-clavulanic acid, 11 (92%) and nitrofurantoin 10 (83%). No resistance against ciprofloxacin, levofloxacin, and gentamycin was exhibited by other isolates and there was also low resistance against tetracycline 1 (8%), ertapenem 1 (8%) and imipenem 3 (25%). Overall, 145 (82%) of the isolates from humans were multi-drug resistant, 65(40%) were ESBL producers and 22 (12.5%) were carbapenemase producers. *E.* *coli* alone contributed 46 (26%) to ESBL prevalence.

**Table 3:** Antibiotic resistance patterns of bacteria isolated from humans and cattle to selected antibiotics.

Drugs	Human isolates			Cattle isolates		
	*E.* *coli* (n = 133), N(%)	*K.* *pneumoniae* (n = 31), N(%)	Others (n = 12) N(%)	*E.* *coli* (n = 84), N(%)	*K.* *pneumoniae* (n = 22), N(%)	*Others* (n = 14) N(%)
Ampicillin	113 (85)	IR	12 (100)	67 (80)	IR	14 (100)
Amoxicillin-clavulanic acid	81 (60)	27 (87)	11 (92)	56 (67)	18 (82)	11 (79)
Cefazolin	130 (98)	29 (94)	12 (100)	81 (96)	20 (91)	13 (92)
Cefuroxime	63 (47)	23 (74)	12 (100)	41 (49)	14 (64)	12 (86)
Ceftazidime	48 (36)	18 (58)	8 (7)	30 (36)	11 (50)	7 (50)
Ceftriaxone	47 (35)	18 (58)	7 (58)	30 (36)	11 (50)	6 (43)
Cefepime	47 (35)	19 (61)	7(58)	30 (36)	12 (55)	5 (36)
Ciprofloxacin	5 (4)	1 (3)	0 (0)	1 (1)	4 (18)	0 (0)
Levofloxacin	5 (4)	1 (3)	0 (0)	2 (2)	4 (18)	0 (0)
Gentamycin	2 (2)	0 (0)	0 (0)	0 (0)	0 (0)	1 (7)
Tetracycline	15 (11)	10 (32)	1 (8)	14 (17)	9 (41)	1 (7)
Nitrofurantoin	28 (21)	13 (42)	10 (83)	27 (32)	11 (50)	7 (50)
Imipenem	5 (4)	2 (7)	3 (25)	4 (5)	3 (14)	3 (21)
Ertapenem	12 (9)	4 (13)	1 (8)	10 (12)	2 (9)	2 (14)
Cotrimoxazole	96 (72)	14 (45)	4 (33)	44 (52)	8 (36)	4 (29)

*IR* Intrinsically Resistant, *Others* *Klebsiella* *oxitoca,* *Serretia* *fonticola,* *Kluyvera* *ascorbate,* *Enterobacter* *cloacae,* *Enterobacter* *aerogenes*).

The *E.* *coli* isolated from cattle exhibited high resistance to cefazolin, ampicillin, and amoxicillin-clavulanic acid (96, 80, 67) %, respectively whereas low resistance to ciprofloxacin levofloxacin and imipenem was noted (Table 3). None of the *E.* *coli* isolated from cattle were resistant to gentamicin. *Klebsiella* *pneumoniae* of cattle origin was mostly resistant to cefazolin, amoxicillin-clavulanic acid, and cefuroxime (91, 82, 64) % respectively but showed low resistance to ertapenem, imipenem, ciprofloxacin, and levofloxacin. None of the *Klebsiella* *pneumoniae* isolates from cattle was resistant to gentamicin. All other organisms (Table 2) isolated from cattle were resistant to ampicillin and highly resistant to cefazolin, cefuroxime, and amoxicillin-clavulanic acid (92, 86, 79) % respectively. None of the other isolated bacteria was resistant to ciprofloxacin and levofloxacin and most of the other antibiotics showed low resistance patterns (Table 3). Multi-drug resistance was detected among 97 (81%) of the cattle isolates, 44 (40%) were ESBL producers and 18(15%) were Carbapeneamse producers. *E.* *coli* alone accounted for the majority, 30 (27%) of ESBL producing isolates from cattle.

## In-depth interviews

### Demographic characteristics of the study respondents

A total of 12 in-depth interviews were conducted with key informants age between 24 and 50 years from different professional backgrounds handling antibiotics. All our respondents consented to be audio-recorded. The educational background of the respondents ranged from high school certificates to Master’s degrees. The qualifications included a Masters in veterinary medicine, Masters of Science in Nursing, Diploma in Nursing, Certificate in nursing, Nursing Assistants who were high school certificate holders trained on the job to do nursing work, Bachelor of Business administration, Bachelors of Community development and Advanced certificate of education. We did not find any participant with a Bachelor of Medicine for our interview because most of the community rural pastoral community health infrastructure was left to the lower qualified staff. Even when one of the clinics in the community was owned by a qualified doctor, it was run by a nursing assistant who he trained on the job.

### Themes

A total of six key themes relating to key informant views on antimicrobial resistance were identified using thematic analysis. In Table 3, we summarized all the six key themes before writing the theme in depth.

### Key informant opinions and perceptions on Antimicrobial resistance

Respondents reported several livestock diseases affecting humans and pastoralist communities in Kasese district for which antibiotics were commonly used. All the four key informants dealing directly in veterinary medicine (who we call veterinary key informants for the sake of this study) reported that the four most common livestock diseases were mainly tick-borne diseases which include; East Coast Fever (ECF), anaplasmosis, heartwater, and babesia. Even when some of these diseases were viral, antibiotics were used as supportive treatment.

*What the community has known is that these antibiotics are broad-spectrum. So when an animal shows any sign of disease, the mind goes back, runs very fast to give antibiotics without knowing whether the antibiotic will be effective or not and what disease it is. The first aid is an antibiotic; that is the practice they have developed over time (KII veterinary pharmacist, Kasese town). *

One of the respondents also reported occasional outbreaks of other notifiable diseases that kill a lot of animals.

*Now in this place the common livestock diseases for which we use antibiotics are tick-borne diseases and the most notorious is Filariosis which is East Coast Fever, ECF but there are also outbreaks of notifiable diseases like Contagious Bovine Pleuropneumonia (CBPP), Foot-and-Mouth Disease (FMD), and cases of lumpy skin diseases. The most recent outbreak reported was Peste des Petits Ruminants (PPR) in goats and sheep which led to the death of several hundreds of goats and sheep before it was controlled by vaccination (KII Veterinary officer, Kasese district).*

On the human medicine side, seven out of eight respondents mentioned malaria, bacterial infection, urinary tract infections, respiratory tract infections, and typhoid as the most common diseases treated with antibiotics.

*“Mostly we have people with a cough. Everybody is coughing, everybody has stomach pain. These we sell to them. at least the antibiotics are helping us to manage the problem”. “If you refuse to sell to them, they leave your place and buy from another place” (KII Nurse Katwe-Kabatooro).*

### Common drugs used in humans and animals

Respondents reported several livestock drugs commonly used among pastoralists in Kasese district. Oxytetracycline with different trade names in different concentrations was reported to be the main antibiotic used by the pastoralist followed by penicillin/streptomycin combination. Sulphadimidine was reported to be used usually when the animal had diarrhea. Respondents also reported common use of de-wormers like albendazole, and liver mysole in different trade names and a few pastoralists had started using ivermectin due to its double effect on worms and ticks though it was perceived to be a very expensive drug.

Acaricides were reported by all the veterinary key informants to be on very high demand. Four classes of acaricides commonly used by pastoralists include amidines, synthetic pyrethroids, the combination amidines, and pyrethroids, the organophosphorus acaricide, however, most pastoralists unknowingly use the same class of acaricide for quite a long time, changing just the brand names (Table 4).

**Table 4:** Inter-host resistance comparison for E. coli isolated from humans and cattle.

Drugs	*Human* (n = 133), N (%)	*Cattle* (n = 84), N (%)	*P* *value*
Ampicillin	113 (85)	67 (80)	0.321
Amoxicillin-clavulanic acid	81 (60)	56 (67)	0.391
Cefazolin	130 (98)	81 (96)	0.565
Cefuroxime	63 (47)	41 (49)	0.836
Ceftazidime	48 (36)	30 (36)	0.955
Ceftriaxone	47 (35)	30 (36)	0.955
Cefepime	47 (35)	30 (36)	0.955
Ciprofloxacin	5 (4)	1 (1)	0.261
Levofloxacin	5 (4)	2 (2)	0.576
Gentamycin	2 (2)	0 (0)	0.426
Tetracycline	15 (11)	14 (17)	0.256
Nitrofurantoin	28 (21)	27 (32)	0.067
Imipenem	5 (4)	4 (5)	0.718
Ertapenem	12 (9)	10 (12)	0.493
Cotrimoxazole	96 (72)	44 (52)	0.003
MDR	10 5(79)	67 (80)	0.885
ESBL	46 (35)	30 (36)	0.865

There was no significant difference in resistance to particular drugs between the human and cattle isolates except for Cotrimoxazole (*P* < 0.05).

*Pastoralists don’t know they are using the same drug so eventually, they begin complaining that the drugs are not working. They say that the drugs are fake (bicupuli) when the problem is lack of knowledge of whatever they are using (KII Veterinary officer, Kasese district).*

On the human medicine side, many antibiotics were mentioned as commonly used in the pastoralist communities (Table 5). Though a long list of antibiotics was reported to be in common use in this community, the people selling or prescribing them were mostly of low medical cadre with a number of the key informants (custodians of antibiotics) practically struggling to pronounce the names of the antibiotics.

**Table 5:** Themes identified during the analysis.

Themes	Subthemes		
(1) Common diseases managed with antibiotics	Cattle	East coast fever (ECF) Anaplasmosis Heartwater Babesia Respiratory tract infections	
	Humans	Malaria Bacterial infections Urinary tract infections Respiratory tract infections Typhoid Ear, Nose and Throat infections Wounds	
(2) Common drugs consumed	Cattle	Oxytetracycline in different concentrations and brands Penicillin and streptomycin (Penstrep) Sulphadimidine Ivermectin Trypanocidals	
	Humans	Amoxiclav Ampiclox Ciprofloxacin Benzylpenicillin Amoxicillin Ceftriaxone	Cotrimoxazole Dexamethasone Metronidazole Ampicillin Gentamicin Chloramphenicol
(3) Awareness of antimicrobial resistance	Awareness	Many people are aware No local term for antimicrobial resistance We have not talked at length about antimicrobial resistance with the pastoralists People do not know that AMR is there but they just see things failing and they blame the drugs that they are counterfeit	
	Source of information	Patients who have not improved on medication Experience	
(4) Drivers of antibiotic resistance		Poverty where people cannot buy full dose No laboratories for culture to confirm resistance Inadequate knowledge of health workers People do not take full dose even when it is there, they leave medicine as soon as they improve Not following instructions Fake drugs on the market	
(5) Surveillance and monitoring		It’s not done Not heard of any	
(6) Proposed intervention		Improve testing services Stop quacks from selling medicine Minimize drug stock-outs in government units Provide charts for antimicrobial resistance Bring new drugs	

### Awareness of antimicrobial resistance

Pastoralists were reported to have limited awareness of antimicrobial resistance and they perceived that treatment failures in animals were due to rampant counterfeit medicines on the market. Most of the veterinary drug shop operators reported being aware of the problem though many could not correctly define antimicrobial resistance.

There was limited awareness about antimicrobial resistance among operators of human medicine clinics and drug shops. Generally, the community bought antibiotics and if they felt the antibiotic did not work they, would go and change to a stronger one, however, educated members of the community were reported to prefer obtaining a prescription from the hospital and then go and buy from the local drug shops and pharmacies since there were often stock outs of drugs in the hospitals.

*Those people who are learned don’t want antibiotics, when it comes to antibiotics, they go to the hospital for a prescription. The information I have received from my patients is that when they start on antibiotics and they take them for a long time, they become addicts. Next time they buy them and take, they will not be treated, for example, somebody buys ampicillin today, tomorrow ampicillin, the next day ampicillin, when he gets small sickness, that ampicillin will not help him because it is already used to the body (KII Drug shop attendant Kinyamaseke).*

One of the respondents interviewed was a masters’ degree nurse who stated clearly that awareness of antimicrobial resistance was not a concern for nursing as a profession.

*You would get that information clearly from the clinician, mostly this would be for the clinicians but I am not a clinician. I am a nurse and not a nurse working in health facilities so you might not get the proper information (KII nursing officer Kasese district).*

### Drivers of antibiotic resistance

Several drivers of antimicrobial resistance were identified by the respondents (Table 5) most of who associated the problem to poverty, illiteracy, lack of veterinary personnel, and ignorance among the community and the low-qualified medical staff serving the community. One operator of a human medicine clinic reported frequently giving antibiotics as first aid because people had no money to buy full doses.

*The drugs are expensive so when the patient says he has no money, you give him an injection for the little money he has, but it is not enough to complete the dose and you have nothing to do for him, you just give for first aid (KII nursing assistant, Nyakatonzi) .*

*The first problem is the patients, who abandon their medication after feeling well, especially in the middle of a dosage. Secondly, the stock-outs of drugs from facilities which makes patients take underdose. Clinical treatment is also a cause of this problem but at a very small scale because chances are equally high that the right medication would be offered to the patients but the practice of sharing one dose of medication among family members is also common (KII nursing officer, Katwe-Kabatooro).*

Three out of eight human medicine practitioners reported that some families shared a drug dose among family members to treat the different infections which are not sensitive to these drugs, hence increasing the problem at hand. Also, patients reportedly did not follow treatment schedules.

*The patients use these drugs badly. You tell the person to come in the evening for an injection, but he comes early in the morning. You tell him again to come in the evening he still comes in the morning. They do not keep time. That is the problem I have seen and it can cause resistance (KII Clinical officer, Hima sub-county).*

Frequent stock out of medicines in the government facilities was reported to be a common problem which the community adapted to by sending children to the public health facilities with false complaints to get several doses of antibiotics and antimalarial to store at home for future emergencies.

Pastoralists were reported to prefer treating their animals because veterinary doctors were few and very expensive.

*one of the reasons why this problem is coming up, pastoralists don’t seek prescription. They do it themselves. So whatever they land on in the drug shops is what they go and use. And even the choice depends on the money they have. So the availability of money also has played a role in this. Then, the treatment done by the pastoralists depends on what the farmer feels. If the farmer feels the animal is fine, he stops medication without completing the dose and stores the rest of the medicine for future use. Drugs were mostly stored inappropriately in the ceilings, metallic boxes, and others place the pastoralists deem fit (KII Veterinary officer Kasese district).*

Polypharmacy was commonly practiced in community pharmacies, clinics, and drug shops due to lack of laboratories in the community health facilities, and antimicrobial resistance was only suspected when the patients returned to the health facility without improvement.

One of the respondents cited political interference as a driver for antimicrobial resistance.

*Now like for us who carry out meat inspection, you find the animal is injected, the oxytetracycline is still visibly present, but the animal has been slaughtered. So when you are inspecting, you find injection sights. When you cut off that piece of meat you see the drug dripping from the meat. You can imagine how many people are going to be affected when they consume that. People are administering these other drugs, trypanocidal. When an animal dies they slaughter it and still eat the meat. When you arrest them, the politicians come and say, ‘leave our people (KII Veterinary officer, Kasese district).*

### Surveillance and monitoring

All the 12 respondents reported that there were no interventions in place to minimize the problem of antimicrobial resistance both in humans and in animals. Drug inspectors often visited drug shops and clinics but their main concern was cleanliness and smart display/arrangement of drugs. Most operators of drug shops admitted to selling antibiotics and the drug inspectors were aware but only advised them not to display antibiotics on the shelves.

*I do not see any intervention because things are being done the way they were being done. Pastoralists still go and do whatever they want to do. I have not seen any intervention in livestock to fight antimicrobial resistance (KII veterinary shop owner, Kasese town).*

Like for Septrin and metro; last time they told us that when we sell septrin and Flagyl, we shouldn’t display them on the shelves (KII drug shop attendant, Kinyamaseke ward).

### Proposed intervention

Some interventions were proposed by the respondents. All the respondents proposed the sensitization of the community as a key intervention and all people who handle antibiotics and the general public should be sensitized.

*I told you that if you train us it can reduce. If you provide us with charts about antimicrobial resistance, different charts hanged in different places, in the clerking room, dispensing room, we can try to adjust. And train the VHTs to tell the people in the communities about the resistance of the drug. You know some people are boozers and they end up becoming resistant. So if you communicate to people, they tend to adjust but through training of health workers. First, train us and we train the community (KII nurse Nyakatonzi health center II).*

We have Class C drug shops; they don’t bother telling us about antibiotic resistance. The inspectors mind about cleanliness and the arrangement of drugs. They told us we can use antibiotics but hide them and not display them on the shelves (KII drug shop attendant, Nyakotonzi).

Some proposed closing down clinics and drug shops operated by unqualified personnel while others proposed that the government should initiate and strengthen surveillance and monitoring of antibiotics at all levels.

*They don’t give them the right dose, even you know how Uganda operates, those drug shops you see, the people there are not real medical people. Those who are selling the drugs may be trained on the job. They just give antibiotics even when they give it in the wrong doses. If somebody was to take it for five days, they will give for two days (KII Clinical officer Hima).*

All the respondents cited awareness campaigns among community health workers and the general population as an important component of the prevention and surveillance strategy for antimicrobial resistance coupled with equipping health facilities with the necessary diagnostic equipment.

*I think they should improve the method of telling us, the drug dealers and sellers in drug shops, that we should be referring these people to hospitals because we are the first people these patients come to before they go to health centers. So we are the ones to tell them. So they should tell the drug sellers to be referring people to hospital (KII drug shop attendant, Kinyamaseke ward).*

*I think after sensitization, facilities should be well equipped such that when people visit these health facilities, they will find the equipment available. But if the sensitization is not followed by the adequate equipment of these facilities, it will all be for nothing however much this information would be very well disseminated or even printed on posters. Besides, if this same information disseminated in the sensitization sessions is extended to radio stations, it would help to ascertain the seriousness of this information (KII nurse Katwe-Kabatooro).*

## Discussion

There was generally low knowledge and awareness on antimicrobial resistance in the pastoralist community of Kasese district and this may be one of the most important contributors to inappropriate use of antibiotics in this community leading to the emergence of multi-drug resistant bacteria. A majority of the community members 74 (78) did not understand the meaning of antimicrobial resistance. A misconception that antimicrobial resistance occurs when your body becomes resistant to antimicrobials and they no longer work as well, was common and this could be as a result of low literacy levels in this community. A majority of our respondents 59 (62%) either had no formal education or ended in primary school and only 6% of the people interviewed had attained a tertiary level of education. Though there was no statistically significant relationship between education level and knowledge/awareness of antimicrobial resistance in our study, in other studies, knowledge, and awareness of antimicrobial resistance was attributed to lack of formal education^12,13^. Awareness campaigns on antimicrobial resistance through effective education and communication coupled with the inclusion of the wider community are important and they constitute the major objective of the Global Action Plan on Antimicrobial Resistance^14^.

Many key informant respondents, 9 out of 12 agreed that antimicrobial resistance was an important issue affecting the pastoralist communities. All the key informant respondents reported that the practice of over the counter purchase of antibiotics was common and attributed this to the challenge in accessing qualified prescribers both for veterinary drugs and human medicine. Whereas some of the clinics in the community were owned by qualified medical doctors, they preferred to employ low cadre medical personnel to run them. The reason for this is that employing a highly trained person required a sound financial position. Previous literature has reported the crucial role of sound financial position to maintain a high quality of service^15^. Most of the respondents obtained drugs from the community drug shops and private clinics without consultation and mostly in sub-optimal doses. Respondents mostly depended on their experience, and advice from friends, family, or neighbors similar to the findings of another study that found that people preferred obtaining antibiotics and taking them basing on their knowledge or guidance from friends and family^16^. Improving the availability of qualified prescribers through deploying more qualified staff in the community health facilities can increase the number of appropriate prescriptions of antimicrobials obtained by the community members. In Kasese district, like other districts in Uganda^17^, only one veterinary officer who was mostly taken up with administrative roles and hardly reached by pastoralists due to his busy schedule was employed. Employing more veterinary officers in Kasese district can improve their availability to the service of the pastoralist communities and hence improve on judicious use of antimicrobials directly by treating animals and indirectly through training and sensitization of the pastoralists.

There were no guidelines found in the dug shops and clinics visited and most prescribers were using their knowledge. This was also observed by a similar study that found that prescribers in community-based practices were less supported than their hospital counterparts and they end up operating in isolation^18^. Because of the assumption that the risk of generating resistance is greater in the hospital environments that are heavily exposed to antimicrobials, most guidelines are tailored to hospitals^18^. Antimicrobial resistance in the community has been reported in several studies around the world^19–21^ yet interventions and policies continue to focus on antimicrobial resistance in the hospital environments. If these efforts are not redirected to the community, the consumption of non-prescribed antibiotics will continue rising. Whereas the adverse effects of non-prescribed antibiotics are rarely reported, they are likely to be as common as the adverse effects of the prescribed antibiotics. Patient safety issues have rarely been of concern even when inappropriate dosing may result in severe allergic reactions, masking the diagnosis of infectious diseases or death^19^.

Though most of the respondents agreed that people should use antimicrobials only when they are prescribed by a doctor or nurse, there were mostly less trained cadres of nurses and nursing assistants operating drug shops and clinics in this community and one needs to get to the hospital and queue in long lines to get a physician’s prescription. A similar challenge of a high prescriber to patient ratio was observed in a study in Nepal^22^ leaving the less trained cadre as the only alternative source of antibiotic prescription. This presents a dilemma of choosing between judicious use and universal access to effective antibiotics. Other studies have noted that universal access to effective antibiotics is essential for tackling the problem of antimicrobial resistance^23,24^ and millions of people die due to lack of access to effective antimicrobials. The reduction in mortality afforded by effective antibiotics in modern medical practice ranging from cancer therapy, neonatology, intensive care medicine to aggressive surgeries, and organ transplantation procedures are quite enormous^25^ but may soon be short-lived. The policies and guidelines to tackle antimicrobial resistance need to put the underserved communities high. The non-prescribed use of antimicrobials in the community has continued to attract less attention leaving the burden of antimicrobial resistance in the hands of those taking care of their critically ill loved ones in the emergency rooms. The resistance developed due to the abuse of particular antibiotics today will affect the treatment of another individual tomorrow^26^.

Vaccination was generally considered an effective means of tackling antimicrobial resistance by the community and all respondents agreed that regular hand washing was key in minimizing the problem of antimicrobial resistance. Our findings agree with previous studies that report that simple infection prevention strategies including hand hygiene are effective in breaking the chain of infection and controlling the horizontal spread of antimicrobial-resistant organisms^27–29^. Other studies have also supported the fact that vaccination is an effective means of reducing the consumption of antimicrobials and controlling antimicrobial resistance^30–32^. Vaccination against bacterial and viral infections reduces morbidity due to the related infections thereby reducing the prescribing of antibiotics and controlling the development of resistance of bacteria to antibiotics.

Drinking lakeshore water and carriage of MDR bacteria in cattle were significantly associated with carriage of multi-drug resistant bacteria in humans (*P* < 0.05). Lake George and the Kazinga channel play a central role in the daily routine of the pastoralist communities in Kasese district. Apart from being a source of drinking water for the cattle, it is a source of domestic drinking water and other uses including recreational purposes for children and adults (Fig. 2). Water is, therefore, a shared resource among the wild, domestic animals, and humans which play a key role in the transmission of antimicrobial resistance at a human-animal interface. Other studies have also similarly observed that shared environments such as water and soil play a vital role in the transmission of antimicrobial resistance^33,34^. Though the causal pathway is difficult to understand, several studies have noted that antimicrobial resistance can be transmitted from animals to humans^26,35^. Resistant bacteria are constantly shed by the cattle into the environment and surface water, directly contaminating caretaker who in Kasese district are often children. Resistant bacteria can then be transferred through several routes to different environments, humans, and animals^26^. Increased use of antibiotics on the farm is reported to drive the evolution of antimicrobial resistance in animals and bacteria confer similar mechanisms of resistance to the commonly used antibiotics in humans^34,36^. Control of antimicrobial resistance, therefore, requires joint efforts from all stakeholders from human health, animal health, agriculture, aquaculture, finance, environment, and well-informed consumers.

Multi-drug resistance in gut bacteria is a growing global public health concern. In this study, antibiotic resistance testing revealed high resistance patterns against cefazolin, ampicillin, cotrimoxazole, and amoxicillin-clavulanic acid among the human isolates of *E.* *coli* (98, 85, 72, 60) %, respectively. Similar findings were documented by another study^37^ in Korea which also associated resistance of *E.* *coli* strains to ciprofloxacin with resistance to ampicillin, cotrimoxazole, and cefazolin. However, gentamicin, ciprofloxacin, levofloxacin, and imipenem showed low resistance patterns (2, 5, 5, 5) %, respectively similar to other studies^38,39^.

Among the human isolates Klebsiella, resistance was high against cefazolin, amoxicillin-clavulanic acid, cefuroxime, and cefepime (94, 87, 74, 61) % respectively and low resistance patterns against ciprofloxacin, levofloxacin, imipenem, and ertapenem (1,1,2,4) % respectively. Our findings are comparable to those reported in other studies^10,40–42^. None of the Klebsiella isolated from humans was resistant to gentamicin.

*Escherichia* *coli* isolated from cattle exhibited high resistance to cefazolin, ampicillin, and amoxicillin–clavulanic acid (96, 80, 67) %, respectively. *Klebsiella* *pneumoniae* from cattle origin was mostly resistant to cefazolin, amoxicillin-clavulanic acid, and cefuroxime (91, 82, 64) %, respectively. High resistance to cephalosporins among the *E.* *coli* and *K.* *pneumoniae* isolated from cattle has been reported in several studies in Africa and elsewhere^43–46^. Ampicillin and amoxicillin resistance has also been commonly observed in several studies^43,47–49^. Low resistance to ciprofloxacin, levofloxacin, and imipenem among the *E.* *coli,* *K.* *pneumoniae,* and other isolates was observed in this study. Fluoroquinolones and carbapenems are antibiotics of critical importance that are still showing low or no resistance in some studies in Africa and elsewhere^50–52^ but rising resistance is becoming a big concern. The low resistance of bovine gram-negative bacteria against imipenem could be because the carbapenems have not been approved for use in animals so use in food animals is likely to be low and therefore minimal direct selection pressure^51^.

None of the *E.* *coli* and *K.* *pneumoniae* isolated from cattle was resistant to gentamicin. Similarly, low rates of resistance (0.89%) of *E.* *coli* isolated from dairy cattle to gentamicin were reported by a study in Zambia^53^ and less than 10% of the isolates from the dairy cattle from farms in Jordan were resistant to gentamicin^54^. On the contrary, higher resistance to gentamicin was reported from the *E.* *coli* isolated from milk in Bengal-India^43^. The varied prevalence of resistance against gentamicin from different geographical regions could be explained by different antibiotic use patterns in these regions.

Other gram-negative bacteria isolated from cattle were all sensitive to ciprofloxacin and levofloxacin indicating that these antibiotics are still useful in the management of bacterial infections in cattle. We detected a high prevalence of multi-drug resistance among cattle isolates 97 (81%) in this study; higher than the findings of another study in China that reported, 40% prevalence of multi-drug resistance from the cattle isolates and 54% in Germany^55,56^. This difference could be attributed to the difference in antibiotic use patterns in the different geographical locations and the recruitment procedure. In the China study, calves entering the fattening sage that might have not had a substantial exposure to antibiotic therapy were sampled whereas, in our study, we mostly sampled already fattened cattle. Overall, 65 (40%) of the bacteria isolated from humans were ESBL producers and 22 (12.5%) were carbapenemase producers and 44 (40%) of the cattle isolates were ESBL producers and 18 (15%) were Carbapenemase producers. This study also noted that there was no statistically significant difference in resistance to a particular drug except for cotrimoxazole in human and animal isolates of *E.* *coli* indicating a possibility of cross transmissibility (Table 4). ESBL and Carbapenemase production seems to be the primary cause of MDR in gut bacteria. Similarly, other studies have observed a high likelihood of ESBL carriage to contribute to MDR among gut bacteria^57,58^. Surveillance of ESBL and carbapenemase production could be one major and useful step in the control of the spread of MDR bacteria in both humans and animals.

## Conclusions

The prevalence of multi-drug resistance among organisms isolated from both humans and animals was high. Consumption of lakeshore water and carriage of multi-drug resistant bacteria in cattle were associated with carriage of multi-drug resistant bacteria in the human population which suggests a possibility of transmission of multi-drug resistant bacteria between humans and animals.

### Recommendations

To attain optimal health for humans, animals, and the environment, the Global and the National Action Plans need to re-direct the efforts to reduce the burden of antimicrobial resistance from the hospital environment to the community. Investment in improving health care delivery systems in the pastoralist communities will improve the health in this population, reduce hospital admissions, and the number of infections that require antibiotics. Public information campaigns are important to improve the knowledge of pastoralist communities on the dangers of non-prescribed use of antimicrobials and the burden of antimicrobial resistance.

## Data Availability

All data on which the conclusions of this manuscript are drawn is available on request from the corresponding author.

## Abbreviations

AMR: Antimicrobial resistance
ESBL: Extended spectrum beta-lactamase
ECF: East coast fever
CBPP: Contagious Bovine pleuropneumonia
FMD: Foot-and-mouth disease
PPR: Peste des petits ruminants
